# Effect of ultraprocessed food intake on cardiometabolic risk is mediated by diet quality: a cross-sectional study

**DOI:** 10.1136/bmjnph-2020-000225

**Published:** 2021-04-07

**Authors:** Jennifer Griffin, Anwar Albaloul, Alexandra Kopytek, Paul Elliott, Gary Frost

**Affiliations:** 1 Nutrition and Dietetics Research Group, Imperial College London, London, London, UK; 2 Department of Epidemiology and Biostatistics, School of Public Health, Imperial College London, London, UK

**Keywords:** dietary patterns, metabolic syndrome

## Abstract

**Objective:**

To examine the effect of the consumption of ultraprocessed food on diet quality, and cardiometabolic risk (CMR) in an occupational cohort.

**Design:**

Cross-sectional.

**Setting:**

Occupational cohort.

**Participants:**

53 163 British police force employees enrolled (2004–2012) into the Airwave Health Monitoring Study. A total of 28 forces across the UK agreed to participate. 9009 participants with available 7-day diet record data and complete co-variate data are reported in this study.

**Main outcome measures:**

A CMR and Dietary Approaches to Stop Hypertension score were treated as continuous variables and used to generate measures of cardiometabolic health and diet quality. Secondary outcome measures include percentage of energy from fat, saturated fat, carbohydrate, protein and non-milk extrinsic sugars (NMES) and fibre grams per 1000 kcal of energy intake.

**Results:**

In this cohort, 58.3%±11.6 of total energy intake was derived from ultraprocessed (NOVA 4) foods. Ultraprocessed food intake was negatively correlated with diet quality (r=−0.32, p<0.001), fibre (r=−0.20, p<0.001) and protein (r = −0.40, p<0.001) and positively correlated with fat (r=0.18, p<0.001), saturated fat (r=0.14, p<0.001) and nmes (r=0.10, p<0.001) intake. Multivariable analysis suggests a positive association between ultraprocessed food (NOVA 4) consumption and CMR. However, this main effect was no longer observed after adjustment for diet quality (p=0.209). Findings from mediation analysis indicate that the effect of ultraprocessed food (NOVA 4) intake on CMR is mediated by diet quality (p<0.001).

**Conclusions:**

Ultraprocessed food consumption is associated with a deterioration in diet quality and positively associated with CMR, although this association is mediated by and dependent on the quality of the diet. The negative impact of ultraprocessed food consumption on diet quality needs to be addressed and controlled studies are needed to fully comprehend whether the relationship between ultraprocessed food consumption and health is independent to its relationship with poor diet quality.

**What this paper adds**

- WHO has recently highlighted that the processing of foods is often coupled with a decline in the nutritional profile of the diet.
- Classification systems have been created to aid categorisation of foods according to the grade at which they are processed. The most popular system is the NOVA Classification of Foods.
- Studies have shown that foods categorised as ultra-processed are often high in fat and low in fibre.
- High intakes of ultra-processed foods have been linked to increased risk of non-communicable diseases, including cardiovascular disease and some cancers.
- This study demonstrates a deterioration in diet quality and cardiometabolic health with increasing intakes of ultra-processed food.
- The association between ultra-processed food intake and poor health is mediated by diet quality.

## Background

The increase in the world population over the last 100 years has been accompanied by an increase in quality of the food supply. This has been achieved through improved agriculture techniques and the processing of foods which ensure a robust ‘farm to folk’ food supply. The processing of food allows increased storage, safety and nutritional quality of food. Some 75% of food sales worldwide are processed.^1^ By 2050, the world will have to feed 9 billion people.^2^ The demand for food will be 60% greater than it is today. At the same time, the United Nations has set ending hunger, achieving food security and improved nutrition, and promoting sustainable agriculture as the second of its 17 Sustainable Development Goals for the year 2030.^2^ Given the interconnection of the food supply within and between countries it is difficult to see how this can be achieved without processing of food.

Although, food processing is integral to feeding the worlds growing population, the consumption of ultraprocessed foods has been associated with a deterioration in diet quality and non-communicable disease risk.^3–10^ Several classification systems have been proposed to classify foods according to their degree of processing^11–13^ of which NOVA is the most used, and the only one that defines the category of ultraprocessed foods. The NOVA system classifies all foods into four groups: unprocessed food (NOVA 1), processed culinary ingredients (NOVA 2), processed food (NOVA 3) and ultraprocessed food (NOVA 4).^14^ Ultraprocessed foods (NOVA 4) have been defined as industrial formulations of ingredients made up entirely or mostly of substances extracted from foods (fats, oils, sugar, starch and proteins) or synthesised in laboratories from food substrates or other organic sources for example food additives and flavourings.^14^ According to this classification system ultraprocessed foods (NOVA 4) include carbonated soft drinks, sweet or savoury snacks, confectionary, mass produced packaged breads, buns, pastries, cakes, biscuits and desserts, prepacked breakfast cereals, preprepared meals, including pies, pasta and pizza dishes, reconstituted meat and meat products, ‘instant’ soup and noodle dishes as well as many other items.^14^

Within the UK alone 56.8%–63.4% of energy intake comes from food that would be classified as ultraprocessed (NOVA 4).^1 15^ With respect to cardiometabolic health, this classification system has been used to demonstrate a link between ultraprocessed food (NOVA 4) consumption, obesity, type 2 diabetes as well as hypertension and other cardiovascular health outcomes.^6 16–23^ Studies have also shown that ultra-processed foods (NOVA 4) consumption is associated with a diet that is higher in fat, saturated fat and lower in fibre.^8 12 15 24–27^ To our current knowledge, the relationship between diet quality as measured using a dietary index score, cardiometabolic risk (CMR) and ultraprocessed food intake has not yet been explored in a UK adult population. Here, we use the Airwave Health Monitoring Study to explore this association in a UK adult occupational cohort.

## Method

### Data collection

**Figure 1:**
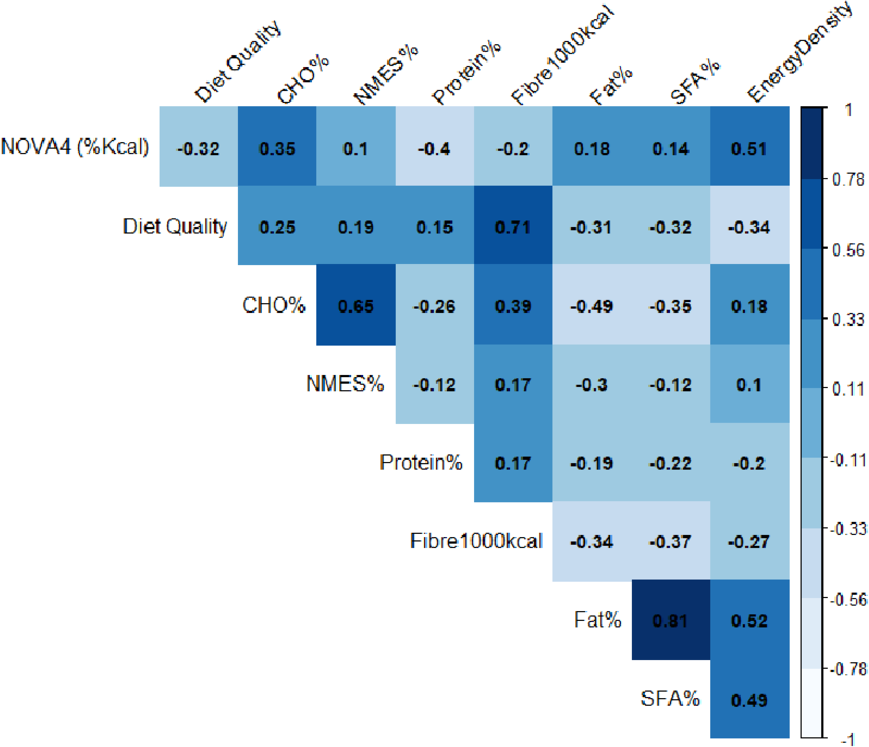
Correlation matrix for the relationship between NOVA 4, diet quality and macro-nutrient intake. The numbers within the matrix indicate the coefficient of correlation between pairs of variables each.

Baseline data from participants within the Airwave Health Monitoring Study were used for the purpose of this study. The Airwave Health Monitoring Study is a longitudinal observational study of members of the police force in Great Britain. Recruitment procedures have been described in detail elsewhere.^28^ For this study, healthy participants with available baseline dietary data as of the end of June 2019 and complete covariate data were included (online supplemental figure, figure 1 participant flow chart). Participants completed 7 days diet diaries and visited an assessment clinic for blood sample collection, blood pressure and standardised anthropometrical measurement.

10.1136/bmjnph-2020-000225.supp1 Supplementary data

### Dietary assessment

Dietary intake was measured using 7-day estimated weight food diaries. Participants were provided with written information and visual aids to assist with portion estimation. Participants were requested to provide any appropriate additional information, for example, method of cooking and names of food brands. Weekly and daily nutritional intake per participant was calculated using the nutritional analysis software Dietplan V.7.0 (Forest field Software, Horsham, UK) which is based on the McCance and Widdowson’s seventh Edition Composition of Foods UK Nutritional Data set (UKN).^29^ Nutritional analysis of the 7-day diet records was conducted by a team of trained nutritionists and dietitians. To ensure consistency and reduce inter-coder and intra-coder errors, coders adhered to a study-specific dietary assessment protocol.

Diet quality was assessed by measuring adherence to the Dietary Approaches to Stop Hypertension (DASH) diet. Higher Adherence to the DASH diet has been associated with reduced CMR.^30^ A DASH index was used to calculate adherence.^31^ This is a 10-point food-based index that assesses diet quality by estimating levels of consumption of different food groups. Points are allocated on meeting targets for consumption of food groups. For example, with relation to fruit and vegetable food group, 10 points are assigned to a participant who meets daily intake as indicated by the index. Participants are allocated points proportional to the target intake. A higher score indicates a higher adherence to the DASH diet and as a result a diet of better quality.

### NOVA classification of UKN composition database

We categorised 6885 UKN/study-specific food and beverage codes into NOVA 1, 2, 3 or 4 group in accordance with the NOVA food classification.^14^ This number represents 100% of the codes used to described dietary intake in this population.

The NOVA classification system requires ingredient information of food/beverage product for product categorisation. As this information was not always available on the nutritional analysis software Dietplan V.7.0, it was assumed that all codes described as ‘homemade’ fall into the NOVA 3 category and those described as ‘retail’ classified as NOVA 4. This decision was taken on the premise that foods described as ‘retail’ are more likely to contain non-nutrient ingredients which are used to identify a NOVA 4 food. There were some exceptions to this rule. For example, when a food or recipe was described as homemade with NOVA 4 food ingredients that is, lasagne homemade made with cook in pasta sauce this food or recipe was assigned NOVA 4. Information attached to food code was used to inform these exceptions. Furthermore, in the case where code description failed to specify whether a food item is of homemade or/retail origin, other information, for example, processing term in food descriptor (canned, tinned) or brand name was taken as an indicator of its origin. Consumption of ultraprocessed was determined by calculating the percentage energy consumed from those foods and beverages categorised as NOVA 4.

### CMR calculation

A CMR score was generated for each participant as previously described.^32^ This score is composed of five components that are indicative of cardiometabolic health. Each component is worth one point. Scoring standards for each component are detailed below. The maximum score is 5 and minimum 0. A person with a score ≥3 is considered at high CMR.

1. Central obesity: waist circumference ≥94 cm—men, waist circumference ≥80 cm—women.

2. Dyslipidaemia: High Density Lipoprotein (HDL) <1.0 mmol/L, men and <1.3 mmol/L women and/or non-HDL ≥4.0 mmol/L and/or prescribed cholesterol lowering medication.

3. High blood pressure: systolic ≥130 mm Hg, and/or diastolic ≥85 mm Hg, and/or prescribed hypotensive medication.

4. Inflammation: High sensitivity Hs-CRP ≥3 mg/L<10 mg/L.

5. Impaired blood glucose control: HbA1c ≥5.7% and/or prescribed medication for glucose control.

### Co-variate data

Covariates included are age in years and body mass index (BMI) kg/m^2^ presented as continuous variables as well as sex, household income (< £25 999, £26 000–£37 999, £38 000–£57 999, £58 000–£77 999, > £80 000) as a measure of socioeconomic class, highest education to date (left before GCSE/equivalent, GSCE/equivalent, A-level/equivalent or Higher), physical activity level (PAL) (high, medium, low), smoking status (current smoker, do not smoke) and dietary misreporting status (under, acceptable, over,) as calculated using the Goldberg cut-off energy intake: basal metabolic rate.^33^

### Patient and public involvement

Public involvement in the Airwave Study comes from engagement with the Police Federation, Home Office, National Police Chiefs’ Council and the police officers and employees involved in the Airwave cohort. The Federation is represented on the Study’s Access Committee, and all three organisations were represented on the Steering Group along with UNISON (representing police staff). The Police Federation work in partnership with us on the enquiries made using the Airwave data set. They have been very supportive in lines of enquiry that provide understanding of lifestyle and the health of the police force.

### Statistical analysis

Both continuous and categorical demographic variables were used to explore sample characteristics. Student’s t-test was used to compare sex differences in continuous data and χ^2^ test for independence to assess differences across categorical variables.

The analysis of variance (ANOVA) test for independence was performed to investigate difference in ultra-processed food intake (NOVA 4) between under, acceptable and over reporters.

We used Pearson correlation to assess associations between ultraprocessed food intake (NOVA 4), diet quality, macronutrient intake (plotted using the ggcorrplot R package) and multivariable linear regression for associations between ultraprocessed food intake (NOVA 4) and diet quality. A backward stepwise approach was taken in building the regression model. The final model included adjustment for known confounders for example, age, sex, smoking status, highest education, level of dietary misreporting and socioeconomic status.

Linear regression modelling was also used to explore the effect of ultraprocessed food (NOVA 4) consumption on CMR independent of diet quality. The final model was adjusted for known confounders: age, sex, DASH Score, level of misreporting, smoking status, PAL, BMI (kg/m^2^), highest education and socioeconomic status.

We conducted causal mediation analysis to investigate whether the effect of ultraprocessed food (NOVA 4) consumption on CMR is mediated by diet quality. To conduct this analysis, we used the R package ‘mediation’ and chose a model-based approach. Mediator and outcome models were adjusted for the following covariates: age, sex, BMI, smoking status, level of misreporting, highest education, PAL and socioeconomic status. To compute the point estimate of the indirect effect, we used the nonparametric bootstrap rather than the quasi-Bayesian Monte Carlo simulation for variance estimation via the boot=TRUE argument, sims=1000. All statistical tests were conducted using R software. Level of significance was set at p<0.05.

## Results

### Descriptive characteristics

A total of 9009 healthy participants with complete 7-day diet record and covariate data were included in this study. The average age was 40.9±9.2 years (60.8% men). The mean percentage of total energy intake from ultraprocessed foods (NOVA 4) was 58.3%±11.6. Sociodemographic, lifestyle and health characteristics are detailed in online supplemental table 1).

### Difference in ultra-processed food intake (NOVA 4) across dietary reporting acceptability categories

More than half of the study population were categorised as acceptable reporters and a significant difference in the distribution of participants was observed across the reporting acceptability categories (p<0.05). The intake of ultra-processed food (NOVA 4) intake increased across reporting categories from participants under-reporting to participants over-reporting. The mean energy intake from NOVA 4 foods was significantly lower in participants categorised under-reporting energy intake than participants with acceptable and over reporting (table 1).

**Table 1:** Ultraprocessed food intake across dietary reporting acceptability categories

		Dietary reporting acceptability categories				
		Under	Acceptable	Over	All	*p value
Total	n (%)	3455 (38.4)	5382 (59.7)	172 (1.9)	9009 (100.0)	<0.001
NOVA 4 (% Kcal)	Mean (SD)	57.2 (12.2)	58.9 (11.2)	61.2 (11.1)	58.3 (11.6)	<0.001

*p<0.05 significant.

### Relationship between ultra-processed food intake (NOVA 4), diet quality and macro-nutrient intake

Pearson correlation analysis (figure 1) shows a positive correlation between intake of ultra-processed food (NOVA 4), intakes of carbohydrate, non-milk extrinsic sugar, fat and saturated fat as well as diet energy density. A negative correlation was observed between ultra-processed food (NOVA 4) intake, protein, fibre and diet quality.

Multi-variable regression analysis was used to further explore the association between ultra-processed food intake (NOVA 4) and diet quality. Both unadjusted (model 1) and adjusted (model 2) model indicate a negative association between ultra-processed food (NOVA 4) intake and diet quality (table 2).

**Table 2:** Association between ultra-processed food (NOVA 4) and diet quality

	Model 1			Model 2		
	Effect, diet quality	SD	P value	Effect, diet quality	SD	P value
NOVA 4 (% Kcal)	−0.212	0.007	<0.001	−0.197	0.007	<0.001

Model 1: crude+age

Model 2: model 1+education+socioeconomic status+level of misreporting.

### Relationship between ultraprocessed food intake (NOVA 4) and CMR

A multivariate regression was conducted to explore association between ultraprocessed food (NOVA 4) intake and CMR. As outlined in table 3, results show a deterioration in CMR with increasing NOVA 4 consumption. After controlling for diet quality, this relationship was no longer significant.

**Table 3:** Association between ultraprocessed food (NOVA 4) and CMR

	Model 1			Model 2		
	Effect CMR	SD	P value	Effect, CMR	SD	P value
NOVA 4 (% Kcal)	0.003	0.001	0.001	0.001	0.001	0.209

Model 1: crude+age+sex+BMI smoking status+education+socioeconomic status+PAL+level of misreporting.

Model 2: model 1+DASH score.

BMI, body mass index; CMR, cardiometabolic risk; DASH, Dietary Approaches to Stop Hypertension; PAL, physical activity level.

### Mediation analysis

**Figure 2:**
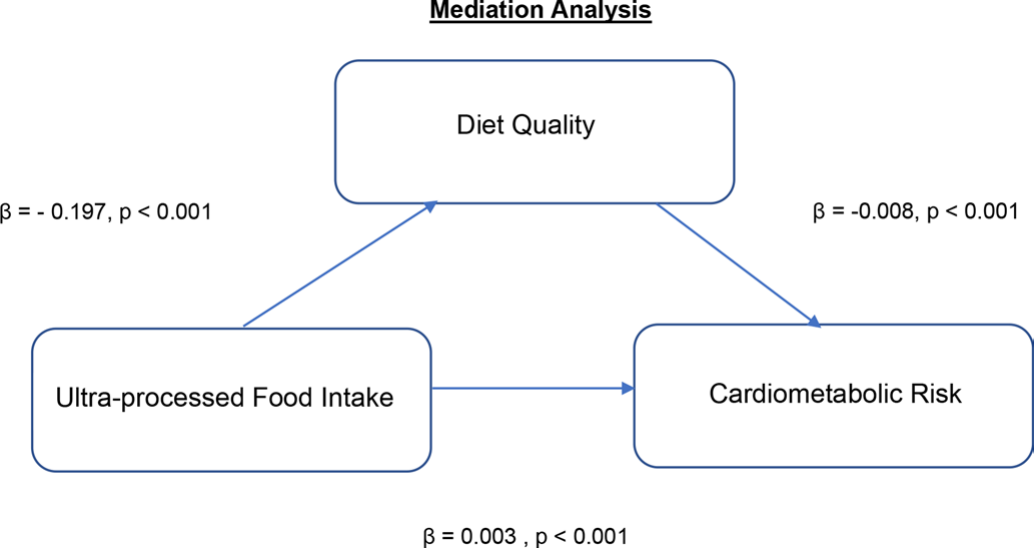
Standard regression coefficients for the relationship between ultraprocessed food (NOVA 4) intake and CMR as mediated by diet quality. CMR, cardiometabolic risk.

The effect of ultraprocessed food (NOVA 4) intake on CMR is mediated by diet quality (figure 2). There is a negative (inverse) association between ultraprocessed food (NOVA 4) and diet quality and between diet quality and CMR. The indirect effect was (−0.197)*(−0.008)=0.0016 (95% CI 0.0011, 0.0022, p<0.001).

## Discussion

Preceding research in this area ranged from studies that examined the relationship between ultra-processed food intake, chronic disease and health,^7 10^ to those that compared the nutrient composition of foods according to grade of processing,^27 34 35^ while, other studies explored variance in dietary composition between those considered high versus low consumers ofultra-processed food.^4 7 10 22–24 36–40^ However, to our understanding, there is no strong evidence to suggest that within a UK population the effect of ultra-processed food intake on disease risk and health is independent of diet quality.

We report here a high proportion of ultraprocessed (NOVA 4) consumption in the Airwave cohort with more than half of total energy intake derived from foods categorised as NOVA 4. Other studies in the UK, France and Canada have reported similar intakes.^1 15 19 41^ These findings are consistent across studies using indirect (food expenditure survey data) and direct (24 hours recall/dietary record data) methodologies to capture food consumption. Our findings reinforce the evidence that the consumption of ultraprocessed foods is increasing.^19^

We also show a negative impact of ultra-processed (NOVA 4) food consumption on diet quality and nutrient composition. Ultraprocessed (NOVA 4) food consumption was positively associated with energy density, intake of fat, saturated fat and non-milk extrinsic sugars but negatively with fibre, protein intake and diet quality. Our findings are consistent with preceding research in this area. Within the UK, high intakes of ultra-processed foods have been positively associated with fat, saturated fat and inversely associated with fibre and protein intakes.^15 41^ In non-UK populations, ultra-processed food consumption has been negatively associated with diet quality in studies using both priori (diet indexes) and non priori approaches (principle component analysis) to measure diet quality.^3 4 17^

We show in a multivariate analysis a deterioration in CMR with increasing ultraprocessed (NOVA 4) food intake. Although this main effect was no longer significant after adjustment for diet quality. Furthermore, findings from mediation analysis show that the impact of ultraprocessed food (NOVA 4) intake on CMR is mediated by diet quality and therefore an effect of ultraprocessed (NOVA 4) food intake on CMR independent of diet quality was not observed. Previous studies in this area have reported a worsening in health and non-communicable disease risk and morality with increasing ultraprocessed food intake.^3–5 7 10 15 18 23 38 39 42 43^ In the few these studies that have adjusted for diet quality a change in effect was not observed.^3–5^

### Strengths and limitations

This study is to our knowledge to first to show that the impact of ultra-processed food intake on CMR is mediated through the deterioration of diet quality. There is also strength in the methodological approach taken in this study. For example, diet quality was determined using a validated diet quality index. This approach is thought to be most advantageous in comparison to posteriori approaches which generate dietary patterns based on available data without a priori hypothesis and these patterns may not represent the optimal.^44^ Furthermore, this study used 7-day diet records to capture dietary intake. This prospective dietary collection method offers detailed information without the limitations associated with recall and Food Frequency Questionnaire (FFQ) techniques. However, it is not short of its own limitations including dietary misreporting a limitation to any subjective measure of dietary.^45^ Studies have demonstrated a positive change in dietary behaviour in light of recording intake. This often means that the intake captured is a false representation of the persons habitual diet.^45^ However, to control for this inherent limitation, the Goldberg cut-off for energy intake: basal metabolic rate equation was conducted for each participant and included in association models to adjust for level of misreporting.^33^ The study also has some other limitations. First, we used cross-sectional data. As a result, a temporal relationship between ultra-processed food intake, diet quality, nutrient composition and CMR could not be assessed. Other shortcomings relate to the categorisation of food codes into NOVA groupings according to the NOVA classification system. Food codes did not always have an accompanied ingredient list which sometimes caused ambiguity to what NOVA group a food code should be placed which may have led to an overestimation in ultraprocessed food intake.

## Conclusion

Food processing is necessary to keep up with feeding the world’s growing population. However, there is strong evidence to show that the consumption of food that has been ultraprocessed is negatively associated with diet quality and health. In this study, we show that the consumption of ultraprocessed food increases CMR through the worsening of the overall quality of the diet. This is the first study in this field to observe this association. The negative impact of ultraprocessed food consumption on diet quality needs to be addressed and controlled studies are needed to fully comprehend whether the relationship between ultraprocessed food consumption and health is independent to its relationship with poor diet quality.

## Data Availability

Data are available in a public, open access repository. All data are available throughout the Dementia Platform https://portal.dementiasplatform.uk/
