# Supplementary material for: Effect of ultraprocessed food intake on cardiometabolic risk is mediated by diet quality: a cross-sectional study
Source: BMJ Nutr Prev Health. 2021 Apr 7;4(1):174–80. doi: 10.1136/bmjnph-2020-000225 (PMC8258022; doi:10.1136/bmjnph-2020-000225)

Supplementary Data

The Effect of Ultra-processed Food Intake on Cardiometabolic Risk is Mediated by Diet Quality.

Authors:

Jennifer Griffin RD. Nutrition & Dietetic Research Group, Department of Metabolism, Digestion and Reproduction, Faculty of Medicine, Imperial College London, UK.

Anwar H. AlBaloul: Nutrition & Dietetic Research Group, Department of Metabolism, Digestion and Reproduction, Faculty of Medicine, Imperial College London, UK.

Aleksandra Kopytek: Clinical Trial Administrator, Nutrition & Dietetic Research Group, Department of Metabolism, Digestion and Reproduction, Faculty of Medicine, Imperial College London, UK.

Paul Elliott MBBS, PhD, FMedSci: Department of Epidemiology and Biostatistics, School of Public Health, Imperial College London, UK

Gary Frost PhD, RD : Nutrition & Dietetic Research Group, Department of Metabolism, Digestion and Reproduction, Faculty of Medicine, Imperial College London, UK

**Supplementary Table 1.** **Study Population Characteristics**					
		**Male**	**Female**	**All**	***p*-value***
**Total**	n (%)	5475 (60.8)	3534 (39.2)	9009 (100.0)	0.031
**Age (y)**	Mean (SD)	42.0 (8.8)	39.3 (9.4)	40.9 (9.2)	< 0.001
**NOVA 4 Intake (% Kcal)**	Mean (SD)	58.4 (11.5)	58.1 (11.8)	58.3 (11.6)	0.298
**BMI Category**					
Underweight (< 18.5kgm2)	n (%)	4 (0.07)	35 (1.0)	39 (0.4)	0.368
Healthy Weight (18.5 – 24.9 kgm2)		1140 (20.8)	1726 (48.8)	2866 (31.8)	<0.001
Overweight (25.0 – 29.9 kgm2)		3045 (55.6)	1227 (34.7)	4272 (47.4)	0.028
Obese (≥ 30 kgm2)		1286 (23.5)	546 (15.4)	1832 (20.3)	0.194
**Ethnicity**					
White	n (%)	5302 (96.8)	3457 (97.8)	8759 (97.2)	0.930
Other		162 (2.9)	71 (2.0)	233 (2.6)	0.684
Prefer not to say		11 (0.3)	6 (0.2)	17 (0.2)	0.887
**Education**					
Left School Before GCSE	n (%)	241 (4.4)	110 (3.1)	351 (3.9)	0.635
GCSE/Equivalent		2095 (38.3)	1235 (34.9)	3330 (37.0)	0.613
A Level/Equivalent or Higher		3139 (57.3)	2189 (62.0)	5328 (59.1)	0.667
**Household Income**					
< £25,999	n (%)	211 (3.8)	534 (15.1)	745 (8.3)	0.009
£26,000 - £37,999		880 (16.1)	769 (21.7)	1649 (18.3)	0.362
£38,000 - £57,999		2459 (44.9)	1156 (32.7)	3615 (40.1)	0.166
£58,000 - £77,999		1378 (25.1)	706 (20.0)	2084 (23.1)	0.448
> £78,000		547 (10.1)	369 (10.5)	916 (10.2)	0.930
*Chi-square Test ~ categorical variables, Students T-test ~ continuous variables. Significance accepted as $p < 0.05$.					

Supplementary Material, Figure 1. Participant Flow Chart

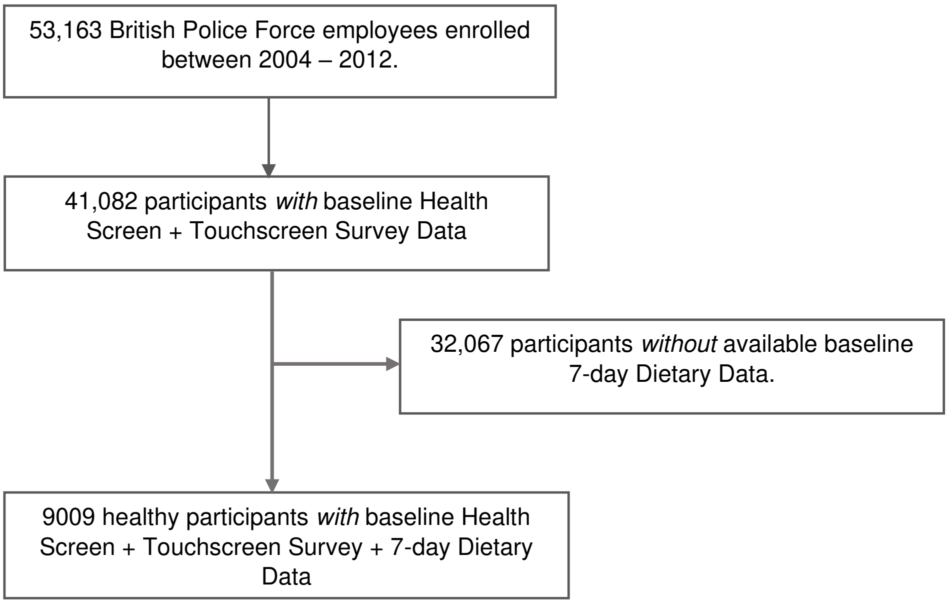

Supplement: Supplementary data [file bmjnph-2020-000225supp001.pdf]
